# How does round goby (*Neogobius melanostomus*) affect fish abundance in the Swedish coastal areas of the Baltic Sea?

**DOI:** 10.1371/journal.pone.0316546

**Published:** 2025-02-24

**Authors:** Rahmat Naddafi, Ann-Britt Florin

**Affiliations:** Department of Aquatic Resources, Swedish University of Agricultural Sciences, Uppsala, Sweden; University of Tehran, IRANISLAMIC REPUBLIC OF

## Abstract

Quantifying the effects of species invasions is particularly challenging, as it requires accurate measurements of the ecosystem before and after the invasion. The round goby (*Neogobius melanostomus*), a highly successful invasive species from the Ponto-Caspian region, has had significant ecological impacts on native communities in the invaded ecosystems. However, there are currently no studies examining the impact of the round goby invasion on the abundance of coastal fish in the Baltic Sea. Using 17–23 years of monitoring data from four areas, we quantified the changes in fish abundance (mostly representing coastal fish indicators and key coastal fish species) associated with the round goby invasion in the Swedish coastal areas. A generalized additive mixed model suggests that round goby invasion will lead to an increase in the abundance of perch, cyprinids, piscivores, and ruffe, while whitefish and flounder abundance will decrease. In addition, the abundance of sprat and herring may not be affected by round goby invasion. Abundance of perch, cyprinids, flounder, perch ( ≥20 cm total length), cod, pikeperch, and pike were increased with water temperature and were decreased with water depth and wave exposure. We observed a decreasing trend in the abundance of whitefish, sprat, and herring with an increase in water temperature and a decrease in water depth. Given the low abundance of several piscivorous species in the Baltic Sea and the role of predators to control exotic prey, reinforcing piscivore populations might be useful for the Baltic Sea ecosystem and regulating round goby populations at a local scale.

## Introduction

Aquatic invasive species pose a serious threat to the ecology, economy, and biodiversity of aquatic ecosystems, in which their effects are difficult to reverse or mitigate [1]. Indeed, quantifying the effects of species invasion is particularly challenging, as it requires accurate measurements of the ecosystem before and after the invasion [2]. The round goby (*Neogobius melanostomus*), a bottom-dwelling fish native to the Ponto-Caspian region, has become a successful invasive species in North American and European aquatic systems [3]. It is a generalist feeder [3–5], has a high reproductive capacity, and is able to tolerate a wide range of environmental factors [3,6–9]. For example, the round goby has a broad thermal tolerance, ranging from −1 to 30°C, exhibits a wide salinity tolerance by inhabiting fresh, brackish, and marine waters, with a reported salinity tolerance of up to 40.5 PSU, and can tolerate very low dissolved oxygen levels, with critical lethal thresholds ranging from 0.4 to 1.3 mg L^−1^ [3,10–12]. It was first observed in the Baltic Sea in the Gulf of Gdansk (the southeastern Baltic Sea) in 1990 [13], and in Sweden in the Karlskrona archipelago in 2008 [14]. This species has now been established in the majority of coastal areas of the Baltic Sea [15].

Since the Baltic Sea has relatively low species diversity and is subjected to many anthropogenic disturbances, it is more prone to biological invasion [16] and can functionally be affected by a new opportunistic invader like the round goby. In the Baltic Sea, the round goby has been shown to compete with native species [17,18], create novel trophic links and energy pathways [5,19], change coastal habitats [20], and negatively impact abundance, taxon richness, and overall body size of benthic invertebrates [21–23]. It has also been shown to be utilized by native piscivorous fish and birds [4,24,25]. In North American and European aquatic systems, both negative and positive effects of round gobies have been reported [3,25–28]. The impact of round gobies on coastal fish populations in the Baltic Sea remains poorly understood. So far, our understanding has been based on indirect inferences drawn from habitat overlap, diet analyses, and experimental studies conducted over short spatial and temporal scales, where fish were exposed to round gobies. Some examples are the study of the interaction between the round goby and flounder (*Platichthys flesus*) [6,18,29], herring (*Clupea harengus*) [30,31], European perch (*Perca fluviatilis*) [6,19,32,33], the northern pike (*Esox lucius*) [14], cod (*Gadus morhua*) [19,34], pikeperch (*Sander lucioperca*) [35], salmonids [36], piscivores including pikeperch, cod, perch, and turbot (*Scophthalmus maximus*) [24,37], cyprinids [38], eelpout (*Zoarces viviparous*) [39], and turbot [40].

To our knowledge, there is no study showing how the abundance of coastal fish species is affected by the round goby invasion in the Baltic Sea or elsewhere. To address this knowledge gap, a model is required that incorporates long-term fish abundance data from before and after the round goby invasion. The model provides valuable information to elucidate the relationship between the round goby invasion and fish populations but also to provide important information to estimate the economic effects of this species and for relevant management action. Furthermore, coastal fish species are key ecosystem components in the Baltic Sea and are therefore used as management objectives within the EU Marine Strategy Framework Directive (MSFD) and the HELCOM Baltic Sea Action Plan (BSAP) [41,42]. The distribution and abundance of local coastal fish communities in the Baltic Sea are influenced by anthropogenic pressures, including eutrophication, habitat deterioration, and fishing, as well as by variations in environmental factors such as water temperature, wave exposure, habitat, and depth [42–45].

Here, we build a model using long-term (up to 23 years) monitoring data on fish abundance in four areas (Muskö and Asköfjärden in the Northern Baltic Proper, Mönsterås in the Western Baltic Proper, and Torhamn in the Bornholm Basin) to quantify the changes in fish abundance (measured as catch per unit effort (CPUE)) associated with the round goby invasion in the Swedish coastal areas. Depending on data availability, we analyzed the abundance of the following fish species/functional groups in response to the round goby invasion and environmental parameters like water temperature, depth, and wave exposure: perch, cyprinids, piscivores, three important piscivores (cod, pikeperch, and pike), perch ( ≥20 cm total length), flounder, herring, sprat, *Sprattus sprattus*, whitefish, *Coregonus maraena*, and ruff, *Gymnocephalus cernua*. The abundance of perch, cyprinids, and piscivores are three important indicators of the environmental status of coastal fish in the Baltic Sea [42]. Perch (a dominant piscivorous species), flounder, pike, pikeperch, and whitefish are key coastal fish species in the Baltic Sea, significantly affecting the structure and function of coastal communities and ecosystems [44,46–49]. Cod, herring, and sprat are commercially important fish species in the Baltic Sea. In addition to flounder, cod is a key species in the more saline western and southern parts and in more exposed archipelago areas [47]. Ruffe is a typical freshwater species that is found in the coastal area, sometimes in high densities [47,50].

## Methods

**Fig 1:**
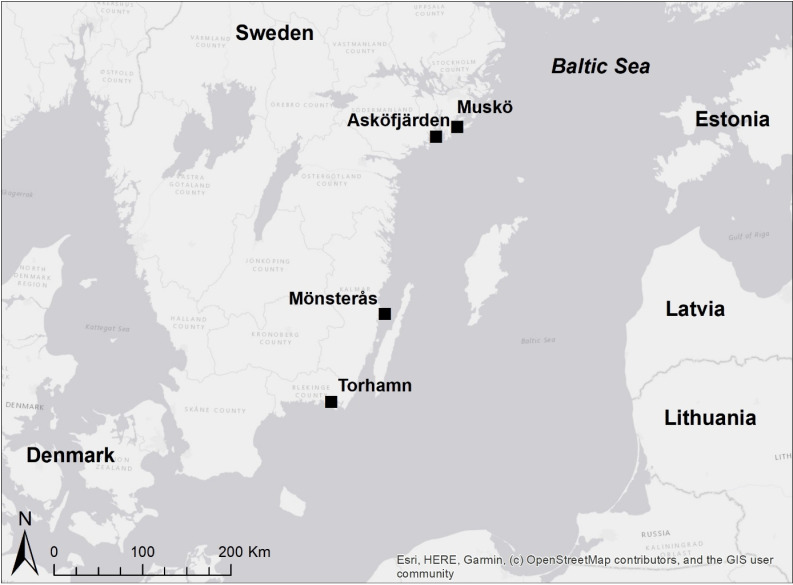
Location of the four areas along the Swedish east coast. The year of first observation of the round goby during the regular fish monitoring program was 2013 in Muskö and Torhamn, 2016 in Mönsterås, and 2018 in Asköfjärden.

The study was conducted using standardized monitoring data of coastal fish from four reference areas with low direct human impact [42,43] at the Baltic Sea (Fig 1). Monitoring in Muskö (8 stations, 2001–2023) and Mönsterås (11 stations, 2007–2023) was conducted using “net series”, where four mesh sections of varying sizes were horizontally linked. In Muskö, the mesh sizes ranged from 21 to 60 mm (i.e., 21, 30, 38, 50, 60 mm) for net series type 1, while in Mönsterås, the mesh sizes ranged from 17 to 30 mm (i.e., 17, 22, 25, 30 mm) for net series type 2 [45,51]. In Asköfjärden (41–48 stations, 2005–2023) and Torhamn (28–50 stations, 2002–2023), the monitoring was done using Nordic coastal multimesh gillnets with mesh sizes of 10, 12, 15, 19, 24, 30, 38, 48, and 60 mm [42,45]. Each survey took place over one to six consecutive nights each year, using either up to 50 Nordic coastal nets or six net series [45,51]. Data were excluded from the analyses if there were signs that the nets had been disturbed by seals, cormorants, or any other unknown factors during the night [45,51]. This was necessary to prevent misestimating fish abundance, as cormorants or seals could have directly lowered catches by removing fish or scaring them away from the nets [45,51]. Catches were registered as numbers of individuals per species and total length class (cm). Only fish >12 cm were included in the analyses, as previous studies showed that fish below this length were not sampled in a representative way [42,45]. The year of first observation of the round goby during the regular fish monitoring program was 2013 in Muskö and Torhamn, 2016 in Mönsterås, and 2018 in Asköfjärden (Database for Coastal Fish – KUL. 2024. Swedish University of Agricultural Sciences (SLU), Department of Aquatic Resources. http://www.slu.se/kul [2024-04-10]). We used this time as a breaking point of the time series for the before and after the round goby invasion in the relevant sampling area. Swedish guidelines were followed concerning the fish sampling within Sweden’s national monitoring program (ethical permit no. SLU.aqua. 2020.5.4-84).

All catches were converted to CPUE as a measure of abundance. CPUE of each species (perch, flounder, herring, large perch, whitefish, sprat, and ruffe) was calculated as the total catch of that species per station and night. CPUE of cyprinids was estimated by the total catch of roach (*Rutilus rutilus*), white bream (*Abramis bjoerkna*), ide (*Leuciscus idus*), rudd (*Scardinius erythrophthalmus*), bleak (*Alburnus alburnus*), bream (*Abramis brama*), tench (*Tinca tinca*), crucian carp (*Carassius carassius*), vimba (*Vimba vimba*), and dace (*Leuciscus leuciscus*) per station and night. CPUE of piscivores was calculated as the total catch of perch (95.7%), cod (2.2%), pikeperch (1.3%), and pike (0.8%) per station and night. Because perch is the predominating piscivorous species in Swedish coastal areas [47], it largely affects the abundance of piscivores. Since we were also interested in the response of other piscivores such as cod, pikeperch, and pike (hereafter CPP) to the round goby invasion, and given that these species belong to the same functional group and may occupy the same trophic level, we combined their abundance data. Pooling the data also ensured a sufficient sample size for our model, particularly since the poor coverage and representation of pike and pikeperch in fisheries-independent coastal fish monitoring programs, along with their low natural population densities, make them typically “data-poor” species [52,53]. Hence, CPUE of CPP was calculated as the total catch of cod, pikeperch, and pike per station and night. Since perch shift to a more piscivorous diet when they exceed 20 cm in size [54], we modelled the response of perch ( ≥ 20 cm total length) (hereafter large perch) to the round goby invasion to determine whether there were any changes in the abundance of these large piscivores.

The variations in CPUE of the coastal fish were explained by water temperature, depth, and wave exposure in a previous study [42]. Thus, we also used these environmental parameters in our model (see below). For data on ambient environmental variables, water temperature was measured at the bottom of each station in connection to the fish surveys, together with information on sampling depth. Information on wave exposure for each station was derived from a digital sea chart using the Simplified Wave Exposure index [55], which combines fetch calculations with wind conditions and also accounts for wave refraction and diffraction effects [42,43].

### Statistical analyses

A Generalized Additive Mixed Model (GAMM) with a negative binomial distribution was used to analyze CPUE of each fish species/functional group in relation to environmental variables as well as categorical factors like the round goby and net type effects. A generalized additive model is a nonparametric method that allows investigating non-linear relationships between dependent and independent variables [56]. This model employs a class of equations called smoothers: algorithms that attempt to generalize data into smooth curves by local fitting to subsections of the data [57]. It has also been successfully applied to analyze environmental effects using both categorical and continuous variables [58].

In our models, the response variable was the mean CPUE of each fish species/functional group, and it was treated as a function of several explanatory variables. The explanatory variables chosen were year (which enables the observation of temporal trends in the data), water temperature, depth, wave exposure, net type, and the presence or absence of the round goby (coded as 0 and 1 for up to 10 years before and after the round goby invasion in each of the areas). Collinearity of covariates was first assessed using the Variance Inflation Factor (VIF) statistic [42,59]. According to Johnston et al. 2018, VIFs of 2.5 or greater are generally considered indicative of considerable collinearity [59]. In the next step, for each fish species/functional group, CPUE was modelled as a function of round goby presence (categorical factor, two levels: absence/presence), net type (categorical factor, three levels), and the smoothing function of continuous variables including year, water temperature, depth, and wave exposure. Area was treated like an independent random effect with a random effect smoother (s4). Hence, the full model applied was:


$$\begin{array}{l} CPUEoffishspecies/functiongroup\sim \;roundgobypresence+\;nettype\\ +\;s_{1}\left(watertemperature\right)\;+s_{2}\left(depth\right)+\;s_{3}\left(waveexposure\right)+\;s_{4}\left(area\right) \end{array}$$

To avoid overfitting the models and to obtain ecologically relevant responses that were easier to interpret, the final models were kept simple [60,61], with the maximum number of knots for each of the smoothers limited to four (k =  4), allowing the smoother to divide the response from each explanatory variable into a maximum of three parts. Because no herring has been caught in Torhamn since 2002, data from this area was excluded in the herring CPUE model. The analyses were conducted with the mgcv package [62] in R software [63]. Statistical significance was accepted at the p <  0.05 level.

## Results

The VIF values were 1.01 for year, 1.85 for water temperature, 1.80 for depth, 1.05 for wave exposure, and 1.04 for the area, suggesting that there was only weak collinearity among covariates. These covariates, in combination with categorical variables (net type and the round goby presence), explained 23%–63% of variations in the abundance of each fish species/functional group (Table 1). The GAMM revealed significant effects of most variables on CPUE of the fish species/functional groups (p <  0.05, p <  0.01, and p <  0.001, Table 1). However, wave exposure and water temperature had only marginal effects on CPUE of herring (p =  0.07, Table 1) and ruffe (p =  0.06, Table 1), respectively.

**Table 1:** Results of the generalized additive mixed model for CPUE of each fish species/functional group. n (number of observations) =  2046 for each fish species/functional group. Only for herring, n =  1191; edf: Effective degrees of freedom.

	Perch			Cyprinids			Piscivores (perch, cod, pikeperch, pike)			Three species of piscivores (cod, pikeperch, pike)			Perch ≥ 20 cm total length		
Deviance explained	27			42			23			44			26		
Coefficients	Estimate	z	P-value	Estimate	z	P-value	Estimate	z	P-value	Estimate	z	P-value	Estimate	z	P-value
Intercept	3.10	36.3	** <0.001**	2.43	21.2	** <0.001**	3.12	38.3	** <0.001**	−1.77	−11.3	** <0.001**	0.98	20.8	** <0.001**
Round goby presence	0.32	3.5	** <0.001**	0.34	2.7	** <0.01**	0.33	3.8	** <0.001**	0.84	4.4	** <0.001**	0.42	4.7	** <0.001**
Net Series type 1	0.44	2.5	** <0.05**	0.73	2.8	** <0.01**	1.21	7.0	** <0.001**	2.51	6.7	** <0.001**	0.48	2.8	** <0.01**
Net Series type 2	−0.03	−0.1	0.894	2.50	9.8	** <0.001**	−0.04	−0.2	0.822	−1.57	−3.5	** <0.001**	−0.56	−5.8	** <0.001**
**Smooth terms**	**edf**	**χ** ^**2**^	**P-value**	**edf**	**χ** ^**2**^	**P-value**	**edf**	**χ** ^**2**^	**P-value**	**edf**	**χ** ^**2**^	**P-value**	**edf**	**χ** ^**2**^	**P-value**
Year	2.33	91.8	** <0.001**	2.64	48.9	** <0.001**	2.80	101.1	** <0.001**	2.96	141.7	** <0.001**	2.91	124.2	** <0.001**
Water temperature	2.65	126.0	** <0.001**	2.81	303.7	** <0.001**	2.80	172.9	** <0.001**	2.78	38.9	** <0.001**	2.93	146.1	** <0.001**
Depth	1.00	80.4	** <0.001**	1.00	137.1	** <0.001**	1.40	56.9	** <0.001**	2.86	97.2	** <0.001**	1.46	94.9	** <0.001**
Wave exposure	2.89	59.9	** <0.001**	2.28	25.5	** <0.001**	2.50	24.8	** <0.001**	2.93	36.0	** <0.001**	2.93	77.6	** <0.001**
Catch area	0.98	40.1	** <0.001**	0.95	19.9	** <0.001**	0.97	42.5	** <0.001**	0.46	0.9	0.171	0.00	0.0	0.673
	**Flounder**			**Herring**			**Sprat**			**Whitefish**			**Ruffe**		
Deviance explained	62			25			63			25			45		
Coefficients	Estimate	z	P-value	Estimate	z	P-value	Estimate	z	P-value	Estimate	z	P-value	Estimate	z	P-value
Intercept	−0.64	−3.8	** <0.001**	2.10	19.4	** <0.001**	2.97	10.0	** <0.001**	−2.57	−16.9	** <0.001**	0.96	5.9	** <0.001**
Round goby presence	−0.29	−1.8	0.06	0.21	1.4	0.165	−0.18	−0.8	0.443	−0.39	−2.0	** <0.05**	0.42	2.6	** <0.01**
Net Series type 1	5.56	13.5	** <0.001**	−1.85	−5.6	** <0.001**	1.43	2.1	** <0.05**	0.23	0.7	0.490	1.81	5.0	** <0.001**
Net Series type 2	1.15	3.1	** <0.01**	0.24	0.75	0.454	7.34	0.9	** <0.001**	−0.7	−1.2	0.216	5.92	15.0	** <0.001**
**Smooth terms**	**edf**	**χ** ^**2**^	**P-value**	**edf**	**χ** ^**2**^	**P-value**	**edf**	**χ** ^**2**^	**P-value**	**edf**	**χ** ^**2**^	**P-value**	**edf**	**χ** ^**2**^	**P-value**
Year	1.00	8.7	** <0.01**	2.96	128.9	** <0.001**	1.00	30.1	** <0.001**	2.92	73.0	** <0.001**	2.85	61.9	** <0.001**
Water temperature	2.61	20.2	** <0.001**	2.96	101.9	** <0.001**	2.56	19.6	** <0.001**	2.73	36.6	** <0.001**	1.00	3.6	0.059
Depth	2.74	35.8	** <0.001**	2.83	47.5	** <0.001**	1.00	128.8	** <0.001**	2.60	83.7	** <0.001**	2.66	189.6	** <0.001**
Wave exposure	2.97	80.5	** <0.001**	2.43	9.7	0.069	2.70	30.4	** <0.001**	2.42	17.1	** <0.001**	2.96	76.8	** <0.001**
Catch area	0.88	7.8	** <0.01**	0.12	1.0	0.136	0.99	155.5	** <0.001**	0.00	0.0	0.816	0.99	136.0	** <0.001**

**Fig 2:**
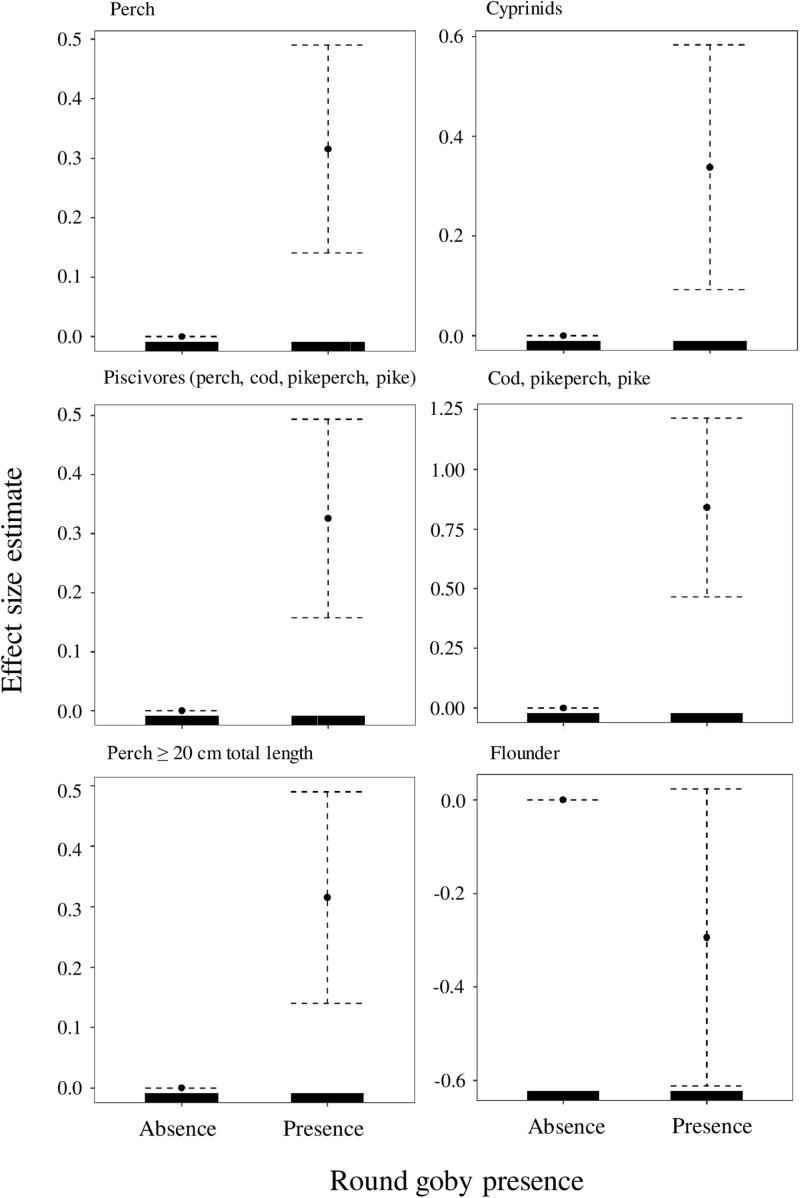
Generalized additive mixed model plots showing partial effects of round goby on CPUE of different fish species/functional groups (perch, cyprinids, piscivores, cod, pikeperch, and pike, perch≥ 20 cm total length, and flounder). Values below zero indicate negative effects of the variable on CPUE. The dotted lines represent the 95% confidence interval. Tick marks on the x-axis indicate the distribution of the observations.

**Fig 3:**
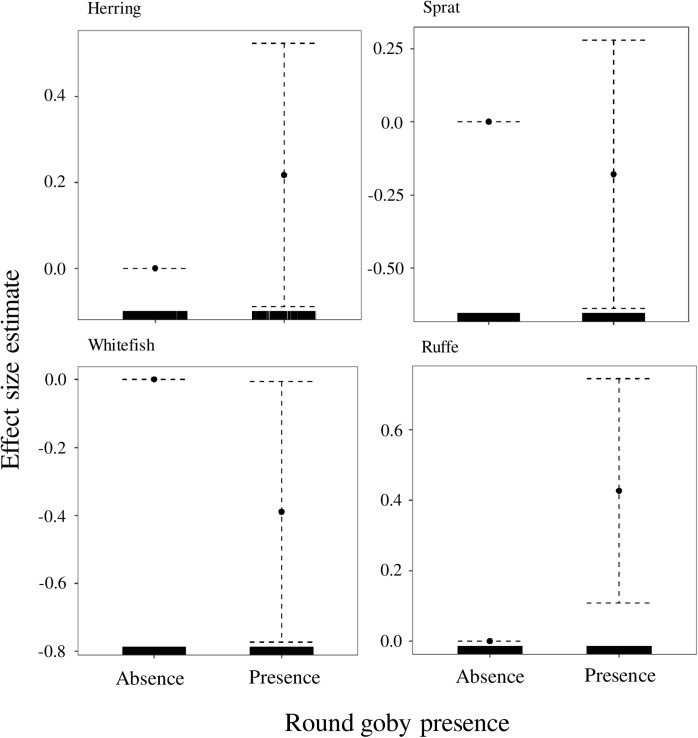
Generalized additive mixed model plots showing partial effects of round goby on CPUE of different fish species (herring, sprat, whitefish, and ruff). Values below zero indicate negative effects of the variable on CPUE. The dotted lines represent the 95% confidence interval. Tick marks on the x-axis indicate the distribution of the observations.

The presence of the round goby had a significant positive effect on the abundance of perch, cyprinids, piscivores, CPP, large perch (p <  0.01, and p <  0.001, Table 1, Fig 2), and ruff (p <  0.01, Table 1, Fig 3), and a significant negative effect on the abundance of flounder (Fig 2) and whitefish (p <  0.05, Table 1, Fig 3), although the effect on the flounder populations was only marginally significant (p =  0.06, Table 1). There were no significant differences in CPUE of herring and sprat in the absence or presence of the round goby (p >  0.05, Table 1, Fig 3).

The catches in the Nordic coastal nets were significantly different from the catches in both net series type 1 and type 2 for CPP, large perch, flounder, sprat, and ruffe (p <  0.05, p <  0.01, and p <  0.001, Table 1). Significant differences were also observed in CPUE of perch, piscivores, and herring between Nordic coastal nets and net series type 1 (p <  0.05 and p <  0.001, Table 1). Net type had no significant effect on CPUE of whitefish (p >  0.05, Table 1). Among net types, the highest CPUE of perch, piscivores, CPP, large perch, flounder, and whitefish was observed in net series type 1 (S1 Fig). In contrast, the highest CPUE of cyprinids, herring, sprat, and ruffe was recorded in net series type 2 (S1 Fig).

The abundance of all fish species fluctuated over time (S2 Fig). There was an increasing trend in the abundance of perch, cyprinids, piscivores, CPP, large perch, and flounder with increasing water temperature (S3 Fig). The abundance of these species/functional groups showed a decreasing trend with increasing depth (S4 Fig). However, the decreasing trend in the abundance of CPP started from depth 12 m and in the abundance of flounder from depth 8 m (S4 Fig). A decreasing trend in abundance of herring, sprat, and whitefish, and, to a lesser extent, ruffe, was observed with increasing water temperature (S3 Fig) and an increasing trend with increasing depth (S4 Fig). Overall, there was a modest decline in CPUE for most fish species/functional groups as wave exposure increased (S5 Fig).

## Discussion

This study demonstrates that the round goby invasion had a strong effect on the abundance of different fish species in our reference areas (Muskö, Mönsterås, Asköfjärden, and Torhamn), stretching from the Northern Baltic Proper to the Bornholm Basin. According to our models, the abundance of perch, cyprinids, piscivores, and ruffe were increased, whereas the abundance of whitefish and flounder were declined following the round goby invasion. CPUE of perch, cyprinids, piscivores, CPP, large perch, and flounder increased with water temperature and decreased with water depth and wave exposure, which corresponds well with the findings of other studies [42–44,64]. In general, among coastal fish communities, percids and cyprinids prefer warmer waters as well as shallower and more sheltered areas of the coastal zone of the Baltic Sea and are favored by higher temperatures [47,65,66]. Likewise, the abundance of adult flounder tends to increase with rising water temperatures [47,67], which lends support to our results.

We observed decreasing trends in the abundance of herring, sprat, and whitefish as water temperature increased and water depth decreased. This is expected, as marine species like sprat and herring, along with some freshwater species inhabiting the Baltic Sea’s coastal areas, such as whitefish, smelt, and sculpins, prefer cooler waters, typically found in deeper regions [47,66]. Furthermore, sprat and herring are pelagic fish, primarily associated with deeper offshore areas.

The positive effects of the round goby on the abundance of perch, piscivores, and the combination of cod, pike, and pikeperch observed in our study are in agreement with expectations based on previous studies showing extensive predation by native fish on the round goby [4,5,24,33–35,37]. For instance, perch primarily feeds on the round goby in the Pomeranian Bay (Bornholm Basin, Baltic Sea) [24,37] and in the Gulf of Gdansk [19,33]. The observed effects of the round goby on CPP, dominated by cod, were particularly strong. The round goby was identified as a new prey species of cod in the southern Baltic Sea [19,68] and in the western Baltic Sea [34]. Using a combination of visual analysis and DNA metabarcoding of stomach contents, Herlevi et al., (2023) found the highest tendency of cod and pike to feed on the round goby in the southern area of the Baltic Proper [4]. Cod and perch consumed fewer crustaceans when the round goby was abundant in the area [4]. The round goby was also found in the stomachs of pikeperch and cod in the Pomeranian Bay [37].

Our model has indicated a decline in the abundance of flounder populations after the round goby introduction into the monitoring areas. This can be explained by competition for habitat and food availability between the round goby and the flounder [5,6,18,32,40]. These two species have been documented to consume similar species and sizes of prey according to stomach contents and stable isotope analyses of wild-caught fish collected from the Gulf of Gdańsk, the southern Baltic Sea [18]. Karlsson et al. (2007) also found a negative correlation between the abundance of these two species in the Gulf of Gdańsk and indicated that round gobies may limit flounder habitat utilization and consequently reduce food availability for flounder [18]. Moreover, Behrens et al. (2022) revealed potentially major overlap (up to 70%) in spatial distribution between these two species in the Baltic Sea [6], and Schrandt et al. (2016) demonstrated direct predation of small flounders ( ≤38 mm total length) by round gobies in a laboratory study [29].

The whitefish population responded negatively to the round goby invasion in our study areas. We do not know the reason for this negative relation, and no studies have, to our knowledge, investigated the interaction between whitefish and the round goby [3,26]. However, other species of salmonids, like lake trout (*Salvelinus namaycush*), have been shown to be negatively affected by the round goby predation on their eggs and larvae [69]. Using an experimental approach, Wallin Kihlberg et al. (2024) observed a delay in salmon spawning in the presence of the round goby and predicted an impairment in the reproductive success of Baltic salmonids if the round goby becomes established in Baltic rivers and streams [36]. Nevertheless, a previous study found an important role of the round goby in the diets of lake whitefish (*Coregonus clupeaformis*) in Lake Michigan during the winter [70].

According to our model, sprat and herring populations are not affected by the round goby invasion. As these clupeids are planktivorous and more dominant in offshore areas in the Baltic Sea [44], they may not compete with the round goby for the food resources. However, Wiegleb et al., (2018) observed small-sized round gobies ( <10 cm) feeding on herring eggs on the German coast (the western Baltic Sea) [31]. Nevertheless, they have suggested that the round goby has less effect on the survival of herring eggs in comparison to other native species owing to a lack of temporal overlap on the main spawning beds and the insignificant amount of consumed herring eggs by large ( >10 cm total length) round gobies [31]. A diet shift from sprat and eel (*Anguilla anguilla*) to the round goby in the cormorants (*Phalacrocorax carbo*) benefited these native species in the Gulf of Gdańsk [71]. Cormorants are known to predate, among other things, on herring, perch, ruffe, and cyprinids in coastal areas in the Baltic Sea [72], and several studies have shown that cormorants consume the round goby [24,71]. However, further investigations are required to evaluate if cormorants switch from native species to the round goby and if this benefits native prey.

Round gobies’ effects on the abundance of cyprinids and ruffe were positive in the current study. It is likely that these fish are released from predation pressure after the introduction of the round goby, a prey that contributes substantially to the diet of piscivores such as cod, pike, and perch [4] and maybe also cormorants (see above). In contrast to our results, Rakauskas et al., (2013) detected the largest trophic overlap between the round goby and ruffe in the Curonian Lagoon, in the southeastern Baltic Sea [73]. They suggested that ruffe might suffer from round goby presence as they likely exploit the same habitat type in the Curonian Lagoon [73]. Skora and Jadwiga Rzeznik (2001) suggested a diet overlap between the round goby and cyprinids like roach and vimba in the Gulf of Gdansk, where round gobies feed mainly on the Pacific blue mussel (*Mytilus trossulus*) [38]. However, in the Baltic proper, the round goby has been shown to feed on hydrobiid gastropods, blue mussels, and several fish species [74], which does not overlap with omnivorous cyprinids. The higher abundance of cyprinids and ruffe after the presence of the round goby in our monitoring areas can also be related to other anthropogenic stressors such as eutrophication, which can be included in future models. The Baltic Sea has been experiencing prolonged nutrient input [47], and cyprinids become more abundant as nutrient levels increase in the Baltic Sea [43,44,47,75]. A high abundance of cyprinids is indicative of eutrophic conditions [43,64,76], but they are also favored by increasing water temperatures and a lack of top-down regulation [49,64,77]. In addition, ruffe catches have been observed to rise along a gradient of productivity [78] and to be more abundant in eutrophic than oligotrophic areas in the brackish water of the Baltic Sea off Helsinki [79].

In general, exotic prey introduced to a new system may experience a release from co-evolved predators or competitors, which may facilitate a rapid population increase in the new environment – the enemy release hypothesis [80,81]. On the other hand, exotic prey that have no evolutionary history with a predator may not be able to recognize or respond appropriately to the risk posed by that predator (the naïve prey hypothesis, [82], but see [83]). In the Baltic Sea, round gobies are preyed upon by several important piscivores, which may explain why it had a positive effect on piscivores in our study. It seems that the positive effect outweighs the negative impacts of the round goby on macroinvertebrates (a food resource for piscivores at earlier life stages) and direct predation on or competition with juvenile piscivores [14,74]. Viable populations of piscivorous species, compared to mesopredators such as cyprinids, are often indicative of an environmental status with few eutrophication symptoms and moderate exploitation [49,76]. It is also important to note that in addition to the temperature [6,9], the likelihood and magnitude of invasion of the round goby and its associated impacts are also dependent on other environmental factors such as salinity [84] as well as propagule pressure. Hence, the influence of the round goby on the fish population should vary across ecosystems [26].

## Conclusion

Using long-term monitoring data on fish abundance in a GAMM model, we predicted that the round goby invasion would result in the increased abundance of perch, cyprinids, piscivores, and ruffe, while the abundance of whitefish and flounder would decline. The perception of this prediction as positive or negative in an ecosystem or societal context depends on the perspective [85], but the MSFD stresses that the impact of non-indigenous species should be minimized. Given a low abundance of some piscivorous species, mainly due to fisheries exploitation and habitat degradation in the Baltic Sea [47,64], and the role of predators to control exotic prey [86], reinforcing piscivore populations might be useful for both the Baltic Sea ecosystem and regulating round goby populations at a local scale (see also [73], but see [27,33]). However, the persistence of changes in fish abundance due to round goby invasion in the Baltic Sea is unclear at this point because several anthropogenic pressures may also influence the abundance of both the round goby and other fish species and interactions between them in the future. Moreover, three coastal fish indicators of environmental status (abundance of perch, cyprinids, and piscivores) also responded positively to the presence of the round goby in this study. We propose that these indicators should be adjusted to local variation in ambient environmental factors such as water temperature, depth, and wave exposure to increase the confidence in the assessment of environmental status [42]. The presence of the round goby has a strong effect on these fish indicators and the key coastal fish species (perch, flounder, cod, pike, pikeperch, and whitefish) in the Baltic Sea. Thus, it might be worthwhile to consider its effects on indicator-based evaluations if this invasive species continues to be widespread and dominant in the Baltic Sea.

## Supporting information

S1 Fig Generalized additive mixed model (GAMM) plots showing the partial effects of net type on CPUE of different fish species/functional groups. (DOCX)

S2 Fig GAMM plots showing the partial effects of year on CPUE of different fish species/functional groups. (DOCX)

S3 Fig GAMM plots showing the partial effects of water temperature on CPUE of different fish species/functional groups. (DOCX)

S4 Fig GAMM plots showing the partial effects of depth on CPUE of different fish species/functional groups. (DOCX)

S5 Fig GAMM plots showing the partial effects of wave exposure on CPUE of different fish species/functional groups. (DOCX)

S6 Dataset (XLSX)
