# Supplementary material for: How does round goby (Neogobius melanostomus) affect fish abundance in the Swedish coastal areas of the Baltic Sea?
Source: PLoS One. 2025 Feb 24;20(2):e0316546. doi: 10.1371/journal.pone.0316546 (PMC11849861; doi:10.1371/journal.pone.0316546)
Supplement: S1 Fig — (DOCX) [file pone.0316546.s001.docx]

**S1 Fig.** Generalized additive mixed model (GAMM) plots showing partial effects of net type on CPUE of different fish species/functional groups. Values below zero indicate negative effects of the variable on CPUE. The dotted lines represent the 95% confidence interval. Tick marks on the x-axis indicate the distribution of the observations.
