# Supplementary material for: How does round goby (Neogobius melanostomus) affect fish abundance in the Swedish coastal areas of the Baltic Sea?
Source: PLoS One. 2025 Feb 24;20(2):e0316546. doi: 10.1371/journal.pone.0316546 (PMC11849861; doi:10.1371/journal.pone.0316546)
Supplement: S4 Fig — (DOCX) [file pone.0316546.s004.docx]

**S4 Fig.** GAMM plots showing the partial effects of depth on the CPUE of different fish species/functional groups. Values below zero indicate negative effects of the variable on the CPUE. The solid line is the smoother and the dotted lines represent the 95% confidence interval. The x-axis represents the value of the model independent variable whereas the y-axis represents the additive contribution of the variable to the nonparametric GAMM smoothing function.
